# Antenna-Coupled Titanium Microbolometers: Application for Precise Control of Radiation Patterns in Terahertz Time-Domain Systems

**DOI:** 10.3390/s21103510

**Published:** 2021-05-18

**Authors:** Liang Qi, Linas Minkevičius, Andrzej Urbanowicz, Andrej Švigelj, Ignas Grigelionis, Irmantas Kašalynas, Janez Trontelj, Gintaras Valušis

**Affiliations:** 1 Center for Physical Sciences and Technology (FTMC), Department of Optoelectronics, Saulėtekio Ave. 3, LT-10257 Vilnius, Lithuania; liang.qi@ftmc.lt (L.Q.); linas.minkevicius@ftmc.lt (L.M.); andzej.urbanovic@ftmc.lt (A.U.); ignas.grigelionis@ftmc.lt (I.G.); irmantas.kasalynas@ftmc.lt (I.K.); 2 Laboratory for Microelectronics, Faculty of Electrical Engineering, University of Ljubljana, Tržaška 25, 1000 Ljubljana, Slovenia; andrej.svigelj@fe.uni-lj.si (A.Š.); janez.trontelj@fe.uni-lj.si (J.T.); 3 Luvitera Ltd., Savanorių Ave. 235, LT-02300 Vilnius, Lithuania

**Keywords:** titanium-based microbolometeric sensors, THz time-domain spectroscopy systems, precise THz mode control, radiation pattern monitoring

## Abstract

An ability of lensless titanium-based antenna coupled microbolometers (Ti-μbolometers) operating at room temperature to monitor precisely radiation patterns in terahertz time-domain spectroscopy (THz-TDS) systems are demonstrated. To provide comprehensive picture, two different THz-TDS systems and Ti-μbolometers coupled with three different antennas—narrowband dipole antennas for 0.3 THz, 0.7 THz and a log-periodic antenna for wideband detection—were selected for experiments. Radiation patterns, spatial beam profiles and explicit beam evolution along the propagation axis are investigated; polarization-sensitive properties under various THz emitter power ranges are revealed. It was found that the studied Ti-μbolometers are convenient lensless sensors suitable to discriminate and control THz radiation pattern features in various wideband THz-TDS systems.

## 1. Introduction

Terahertz time-domain spectroscopy (THz-TDS) is a distinctive example of a wideband electromagnetic radiation system invented more than three decades ago [1]. The THz spectroscopy is very powerful technique for the test and discrimination of different kind of materials [2,3,4]; it can successfully serve for a medical inspection [5,6], security aims [7,8], photovoltaics industry [9,10] as well as food control purposes [11,12].

From the point of view of THz imaging, it displays a possibility for multispectral (colored) THz imaging enabling thus discrimination of different materials without recording their spectral signatures directly [13,14,15] as “colored” images of the THz light contain information on chemical composition, spatial distributions and physical properties of tested objects. One needs to note that the quality of obtained images are highly sensitive to the radiation pattern of scanning beam [16]. This circumstance becomes of a particular importance in real-time inspection and sensing when a high capture rate of images is required, and detectors arrays need to be implemented in the recording set-up using collimated beams. Moreover, even in the case of bulky objects imaging, when extended focus [17] or bifocal (Fibonacci) focusing [18] helps to discriminate the content, control and monitoring of the beam shape along its propagation axis demands of special attention.

High quality images of optoelectronically generated radiation patterns covering 0.1–2 THz range were recorded by room-temperature antenna-coupled α-silicon-based microbolometers array with integrated CMOS read-out electronics [19]. Thin film vanadium oxide (VOx)-based microbolometers arrays were demonstrated as promising tool for label-free detection of reaction of small molecules with proteins [20]. Titanium-based microbolometers (Ti-μbolometers) were found to be useful and convenient instrument in security [21], plastic package inspection and spectroscopic-spatial analysis of materials proving the maturity of THz technology for medicine applications [22].

Very recently, we have demonstrated that optimised room temperature Ti-μbolometers can serve as a convenient and easy-to-use imaging tool to accurately adjust low power THz sources without additional focusing components [23]. Radiation patterns and spatial modes profiles generated by electronic sources, frequency domain spectrometers, optically pumped molecular THz laser were measured and analyzed [23]. In addition, it was shown that these devices exhibiting room temperature sensitivity values of 200 kV/W, noise-equivalent power (NEP) of 20 pW/$\sqrt{\mathrm{Hz}}$, and the response time in the 5 μs range are sensitive and fast enough to monitor precisely coherent THz emitters in optoelectronic time-domain systems.

In this work, we present comprehensive experimental study dedicated to reveal features of Ti-μbolometers for spatial mode control in THz TDS systems. Three lensless Ti-μbolometer sensors with different antenna designs—narrowband dipole antenna for 0.3 THz, 0.7 THz and a log-periodic antenna for wideband detection—are explored in detail. Radiation patterns, spatial beam profiles and explicit beam evolution along the propagation axis was examined, polarization sensitive properties were revealed under different THz emitter power range. It was shown that the Ti-μbolometers do not require additional focusing optics to discriminate spatial mode features in the wideband THz pulse. It was revealed that Ti-μbolometer sensors and their arrays can be employed as easy-to-use imaging instruments in THz TDS systems for accurate optical alignment and quality control of their radiation patterns.

## 2. Materials and Methods

### 2.1. Design and Spectral Characterization of Ti-μbolometer Sensors

**Figure 1:**
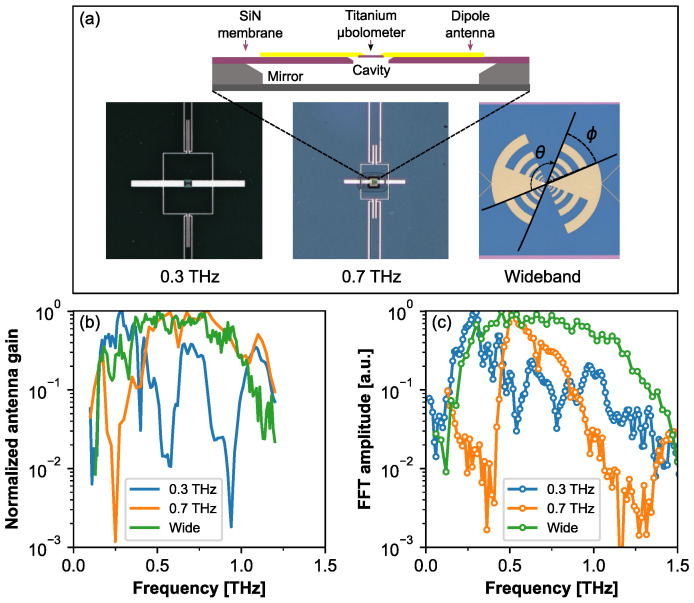
The design and response spectra of antenna-coupled Ti-μbolometers. Top panel (**a**): The design and schematic cross-section of the Ti-μbolometer coupled with different antennas for 0.3 THz, 0.7 THz and for wideband detection (from left to right). Digital photos show top views of Ti-μbolometer detectors coupled with dipole antenna design. The geometry of the titanium bridge amounts to 12 μm length and 2 μm width. The angles θ and ϕ in photo of the wideband detector are defined as sample rotation angle for polarization measurements and the polar angle of antenna fingers, respectively. The design is adapted from Refs. [22,23]. Panel (**b**): Simulated spectra of the normalized antenna gain for the different antenna’s designs. Panel (**c**): Response spectra of the devices were measured using Fourier transform far-infrared spectrometer as described in Ref. [22].

The design of antenna-coupled Ti-μbolometers and their frequency properties are presented in Figure 1. Each microbolometer was fabricated from titanium which was found to be the most suitable for silicon processing technology, temperature coefficient and reliability. The titanium bridge of 12 μm and 2 μm in length and width respectively was electrodeposited on the silicon nitride (SiN) membrane of thickness of 2 μm. The aluminum antenna and interconnection paths of area of approximately 500 μm × 500 μm, were formed around the active part of Ti-μbolometer (see in top of the panel (a) in Figure 1). To reduce the heat capacitance and to increase the speed of operation, the Ti-μbolometer was suspended in the air by etching out the underlying SiN membrane and was coupled either to resonant double-dipole or to wideband log-periodic type THz antenna (see panel (a) in Figure 1) [22,23,24]. Sensitivity of the device was enhanced resonantly by adjustment of a dipole antenna and resonant-cavity design: the back side reflection mirror was placed at the quarter wavelength distance (Figure 1a).

The active gain of each antenna was numerically simulated and is shown in Figure 1b. Detectors’ response spectra at room temperature were also characterized experimentally employing a custom-made Fourier spectrometer. The reference spectrum was registered when interferometer was coupled with a thermo-acoustic Golay cell detector featuring the flat response in THz frequency range. Finally, in order to avoid the influence of emitter and the beamsplitter spectral response the bolometer detected spectrum was divided by the reference spectrum detected by the Golay cell. As it can be seen from the normalized response spectra given in Figure 1c, the resonant Ti-μbolometers designed for 0.3 THz and 0.7 THz show the detection peaks around the frequencies of 0.3 THz and 0.65 THz, respectively. Additionally, at higher frequencies, lobes exhibiting lower amplitudes are observed which most probably come from higher dipole antenna resonances. The detector with log-periodic antenna demonstrates detection bandwidth in the range of 0.25–1.3 THz (Figure 1b). As is visible from Figure 1c,b, the obtained experimental data correlate well with the theoretical predictions.

The sensitivity and NEP values are given in Table 1. Additionally, here the geometrical bolometer antennae parameters such as length *L*, width *W* and polar angle ϕ of antenna fingers of the wideband detector are tabulated.

### 2.2. Experimental Setup of THz Time-Domain System

**Figure 2:**
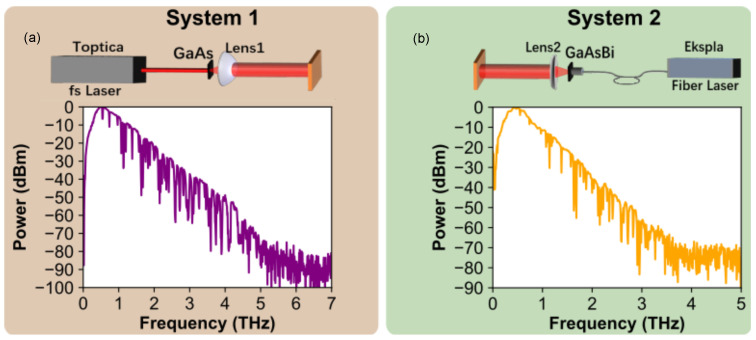
Schematics of THz TDS investigation set-ups and their emission spectra. System 1 (panel (**a**)) is based on femtosecond fiber laser (Femtofiber Pro, Femtofiber Pro, Toptica Photonics Ag, Munich, Germany) delivering pulses of 780 nm wavelength and 90 fs pulse duration pulses to excite THz emission from photoconductive antenna made from LT-GaAs. System 2 (panel (**b**)) consists of femtosecond fiber laser (LightWare FF50, Ekspla, Vilnius, Lithuania) with a center wavelength of 1064 nm and 150 fs pulse duration pulses employed to excite THz radiation from GaAsBi-based emitter. Please note that both THz spectra are decorated by sharp lines of absorption in water vapour.

The optical outputs of two different THz-TDS systems without reference beams schematically shown in the upper parts of Figure 2a,b were characterized using three different Ti-μbolometer designs described above.

In THz-TDS System 1, a femtosecond fiber laser (Femtofiber Pro, Toptica Photonics Ag, Munich, Germany) providing pulses of 780 nm wavelength, 90 fs pulse duration and 150 mW output power at 80 MHz pulse repetition rate was used to excite photoconductive antenna made from 350 μm-thick LT-GaAs wafer. A THz emitter antenna (Teravil Ltd., Vilnius, Lithuania) had the shape of a H-dipole made from Ti/Au with the width of 20 μm and the gap of 50 μm between electrodes. A 8 mm-thick and 12 mm in diameter high resistivity hyperhemispherical silicon lens (Lens 1) with a focal distance of 500 μm was used to collimate the THz radiation. A reference THz spectrum measured with THz detector (Teravil Ltd., Vilnius, Lithuania) is shown in the bottom of Figure 2a. THz detector was a Hertzian dipole type photoconductive antenna made from LT-GaAs with a 6 μm width gap. The emitter exhibits a wide emission spectrum ranging from 0 to 5 THz, as it can be seen from the panel (a) in Figure 2.

In THz-TDS System 2, femtosecond fiber laser (LightWare FF50, Ekspla, Vilnius, Lithuania) with a center wavelength of 1064 nm, 150 fs pulse duration, 60 mW output power and 30 MHz repetition rate was employed. The source of THz radiation was a photoconductive emitter made from LT-GaAsBi (Teravil Ltd., Vilnius, Lithuania) wafer with thickness of 500 μm. For collimation, silicon hyperhemispherical lens (Lens 2) with the same specifications as in System 1 was used. A THz emitter/detector antenna was the H-dipole made of Ti/Au with dipole antenna gap size of 6 μm. The reference THz spectrum measured with THz detector (Teravil Ltd., Vilnius, Lithuania) is given in the Figure 2b. It is seen that it extends up to nearly of 4 THz.

The investigated Ti-μbolometers were mounted on a 3-dimensional *x*,*y*,*z* electric translation stage and mechanical rotation stage. The detector can be moved in the x−y plane for a spatial mode profile detection; separately, a movement along z−axis enables testing the beam profile along the propagation direction. While dipole antenna equipped Ti-μbolometers are sensitive to polarization, rotation with the θ−angle as shown in the (a) panel of Figure 1 allows studying THz beam polarization properties.

The signal of radiation emitted by TDS source was captured by the μbolometer and was read out using the conventional lock-in technique. The reference was taken from a mechanical chopper modulating the femtosecond laser beam at 930 Hz.

## 3. Results and Discussion

The radiation patterns of THz-TDS spectrometers were investigated via five diverse approaches. Initially, spatial modes profiles were examined in x−y plane, i.e., perpendicular to the beam propagation direction within two distances—very close to the THz emitter, placing the Ti-μbolometer at 10 mm from the source; then—recording it in a far-field, as far as 115 mm from the source. The next step was to explore spatial profile features along the propagation direction and to study polarization properties of the devices. Finally, collimating lenses were removed from the emission set-up aiming to illustrate abilities to resolve spatial modes of the emitter without any optical components. The scanning was performed moving the stage at continuous velocity of 25 mm/s with the lock-in averaging constant of 20 ms. The acquisition of 12 mm by 12 mm image took approximately 350 s for the given scanning velocity.

### 3.1. Features of the Radiation Patterns

**Figure 3:**
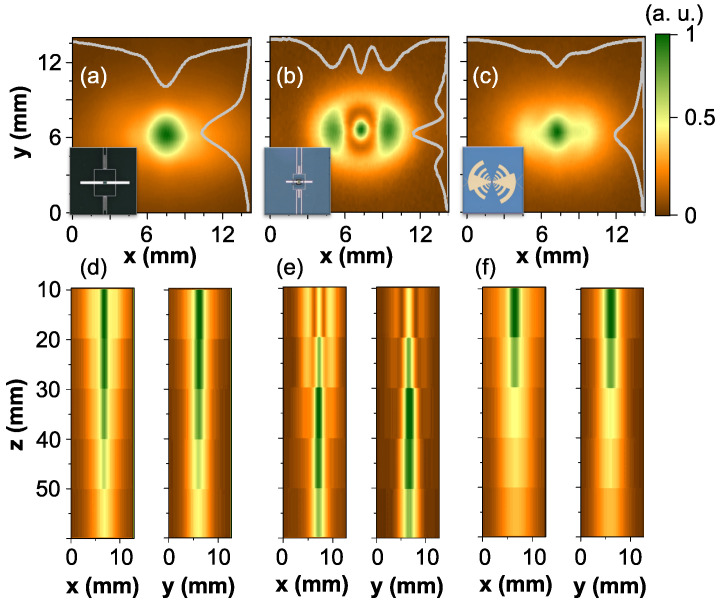
The radiation patterns measured with different Ti-μbolometers placed at z=10 mm position: Panel (**a**)—0.3 THz μbolometer; panel (**b**)—0.7 THz Ti-μbolometer, panel (**c**)—wideband Ti-μbolometer. Grey lines indicate intensity center cross-sections along the relevant axes. Note particular spatial mode profiles clearly resolved by different antenna-coupled Ti-μbolometers. Panels (**d**–**f**) depict x−z and y−z scans from 10 mm to 50 mm along *z* direction for 0.3 THz, 0.7 THz and wideband Ti-μbolometers, respectively.

The radiation patterns scanned in x−y plane in the distance of 10 mm from the emitter lens tip using Ti-μbolometers of three different antenna designs are depicted in Figure 3. The conventional femtosecond Ti-Sapphire system emitting wideband THz pulses in a frequency range of 0.1–2.5 THz with an average power of 1.2 μW served as a THz source. The emitter was based on 500 μm-thick LT-GaAs wafer, the lens parameters are the same as described above. As it can be seen, radiation patterns recorded with different antenna-coupled Ti-μbolometer exhibits different features: 0.3 THz resonant Ti-μbolometer displays the symmetric mode spot (Figure 3a), while distinctive feature of 0.7 THz frequency sensors is strongly expressed side lobes in intensity distribution (Figure 3b). Spatial beam pattern registered by the wideband detector reveals a bright central spot accompanied by well-pronounced side lobes as presented in Figure 3c. The spatial evolution of wideband THz pulses along an optical axis is shown in Figure 3d–f. Results demonstrate that spatial features-enriched beam profile evolves into almost symmetric Gaussian beam at the distance of 50 mm from the lens. This illustrates nicely that the Ti-μbolometer is quite convenient and sensitive enough to characterize the performance of THz-TDS emitter with the hyperhemispherical lens attached. The recorded pattern structure can be understood as follows: at low frequencies a wide central part is formed, while at higher frequencies the central pattern part becomes narrower [25]. Regarding side fringes, their origin is related to internal reflections in the lens, and are emitted in smaller angles (bend closer to the central beam part). Consequently, only the wide central beam part is registered with 0.3 THz frequency sensor, while the 0.7 THz and the wideband μbolometers display the narrowed central part and fringes around it.

A precise optical alignment of THz radiation in TDS spectrometer and monitoring of the beam pattern propagation can aid in improvement of system performance, e.g., by increasing its dynamical range and lowering the noise floor—features which are of particular importance for imaging and spectroscopy. Therefore, the Ti-μbolometers were tested as possible instrument for these aims intending to provide full experimental characterization of THz beams evolution via amplitude distribution across the THz beam in a far-field varying the distance from 5 cm up to 12 cm range from the emitter.

**Figure 4:**
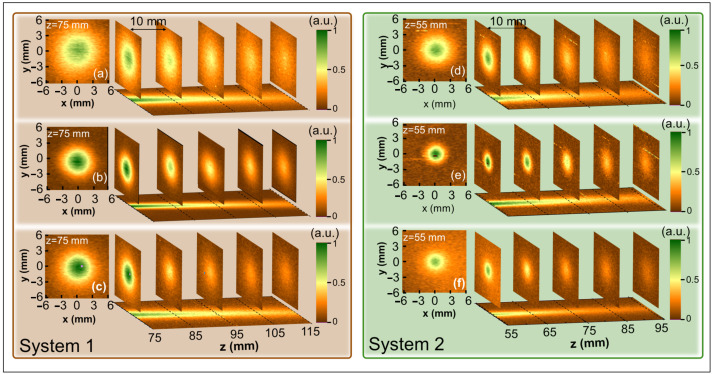
Radiation pattern distributions and beam evolution along the propagation axis recorded by antenna-coupled Ti-μbolometers for two different THz-TDS systems. The signal is normalized to maximum values of the relevant plots. Left panels displays spatial mode profile at starting positions. Note different mode spatial diameter if compare both systems at the same distance from the lens tip. It stems from different thicknesses (350 μm vs. 500 μm) of emitters used in experimental setups. As emitter thickness of System 1 was not well matched to the lens focal distance, it results to a larger divergence of the emitted mode profile. Beam pattern profile evolution was investigated in the range of 75 mm–115 mm and 55 mm–95 mm after the collimating lens in System 1 and System 2, respectively. Distances were selected according to mechanical limitations of geometrical dimensions of the used emitters and detectors. Five transverse mode profiles samples were recorded every 10 mm along *z*-direction for each TDS system. Panels (**a**–**c**)—System 1; panels (**d**–**f**)—System 2. Panels (**a**,**d**)—0.3 THz; panels (**b**,**e**)—0.7 THz; panels (**c**,**f**)—wideband Ti-μbolometer. Please note that the spatial profiles and beam propagation properties of both systems exhibit no essential differences.

The results of investigation for both THz TDS systems are given in Figure 4 starting at 75 mm and 55 mm distances, for System 1 and System 2, respectively. Such starting points were chosen according to the geometrical restrictions in investigated systems. The first feature that attracts attention is different beam spot sizes if compare starting recording data of System 1 and System 2—the beam spot is significantly smaller in the latter case. As was aforesaid, the hyperhemispherical lens of the same design was used to collimate the emitters beams profile. To do it effectively, one needs to place the emitter in its focal point. As the THz emitters in the experimental setups were of different thicknesses (350 μm vs. 500 μm), the observed differences unveil that emitter thickness of the System 1 was not well matched to lens focal distance resulting thus into a larger divergence of the emitted beam mode.

The beam pattern evolution along the propagation axis illustrated via five x−y cross-sections of the mode profile registered every 10 mm is presented in Figure 4. As it can be seen from Figure 4, the investigated pattern does not experience essential changes with the distance. Note highly expressed central part of pattern decaying with the distance and absence of side-lobes in this far-field experiment. The beam is also of small divergence which was estimated Θ≈0.045 rad at 0.7 THz in both experimental systems. Since $\Theta =\lambda /2\pi n\omega_{0}$, where λ is a wavelength, *n* denotes the refractive index and $\omega_{0}$ stands for a beam waist, we can easily find that $\omega_{0}=1.52$ mm for the above given divergence and radiation frequency in System 2.

These experimental observations are in a good agreement with theoretically calculated beam patterns in the distances far from the lens tip [25]. However, one can underline the variation of the central spot diameter of the detected pattern with frequency in both THz TDS systems. The beam profile recorded with the sensor designed for 0.7 THz frequency displays the smallest diameter 5.10 mm in System 1 versus 2.55 mm in System 2, the wideband Ti-μbolometer—slightly larger beam diameter 7.95 mm vs 3.45 mm, while the profile recorded with the antenna-coupled Ti-μbolometer for 0.3 THz amounts to 8.40 mm vs 4.95 mm. The accuracy here is in the order of 0.15 mm. As already mentioned earlier, the beam width at 0.3 THz is the largest because of the longer wavelength and larger dipole antenna dimensions (see Table 1). The wideband Ti-μbolometer results (Figure 4c,f) show the beam spot size which is close to that for 0.3 THz in Figure 4a,d, but with higher intensity of the central part of the beam. Such increase in intensity is governed mainly by the Ti-μbolometer’s responsivity bandwidth (see Figure 1) and by the fact that the lens concentrates higher frequencies in wideband THz emission closer to the optical axis.

### 3.2. Polarization Properties

Features of the presented Ti-μbolometer design enables it to be sensitive to the polarization of the THz radiation—if the sensor axis is aligned in parallel to the THz field electrical component of the incident light, i.e., θ=0 deg—the coupling should be efficient resulting therefore to the maximal heating of the μbolometer’s titanium bridge, and, as a consequence, to the largest signal. Gradual rotation around optical axis (0≤θ≤90 deg) can serve hence as polarization-resolved tool permitting using the Ti-μbolometer to monitor polarization properties in THz TDS spectrometer.

**Figure 5:**
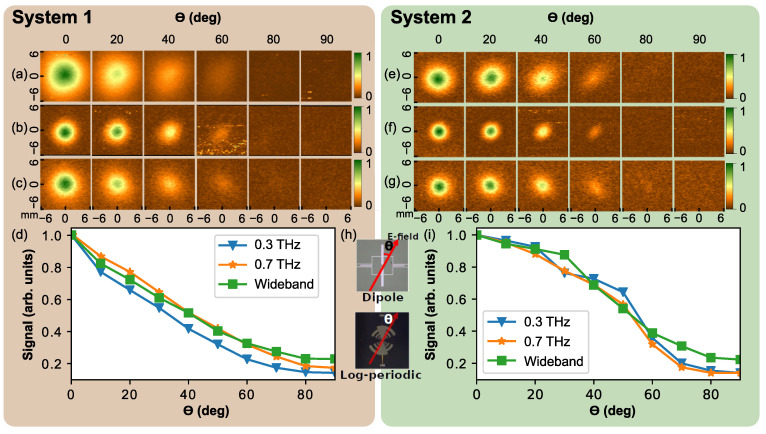
Polarization dependencies of the radiation patterns at various distances along the beam propagation axis recorded by different type of antenna-designs coupled Ti-μbolometers. Panels (**a**,**e**)—0.3 THz; panels (**b**,**f**)—0.7 THz; panels (**c**,**g**)—wideband Ti-μbolometer. Panels (**d**,**i**)—the angular dependencies of the signal at the pattern center normalized to the signal at θ=0 deg, for System 1 and System 2, respectively. Panel (**h**) depicts rotation angle θ with respect to the Ti-μbolometer axis.

Investigations were performed at distances 75 mm and 55 mm from the lens tip (front *x*-*y* scan planes positions as given in Figure 4) in System 1 and System 2, respectively. The obtained polarization results for all the investigated μbolometers are shown in Figure 5. To avoid possible misalignment artifacts, the angular dependencies of signal at the pattern center are normalized to the signal at θ=0 deg and presented in panels (d) and (i) for different THz TDs spectrometers. As is seen, the dependencies decrease gradually with the rotation angle and flatten above 80 degrees, where the signal level approaches the noise floor.

One can indicate that an ellipticity of the beam spot and its smooth decrease down to the noise level is recorded by the Ti-μbolometer along with θ change. This feature indicates that the sensor can be well-suited to monitor polarization state or cross-polarized angular emission patterns [26] of THz pulsed emission—it is rather important issue for the most of common real implementations of THz-TDS spectrometers or THz imaging systems.

### 3.3. The Radiation Patterns and Spatial Mode Profiles Recorded without Collimating Optical Components

**Figure 6:**
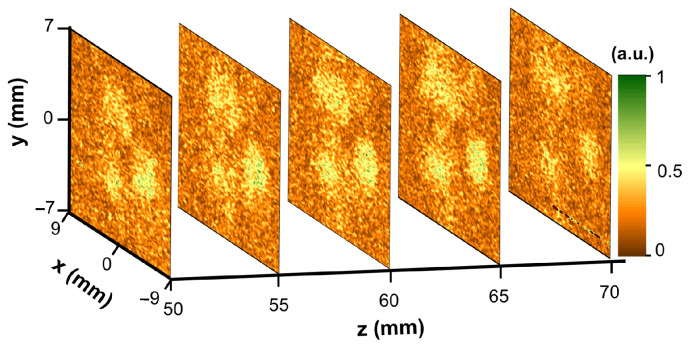
Spatial beam profiles of System 1 recorded by 0.7 THz Ti-μbolometer without collimating lens at different distances along the beam propagation direction. Note different spatial antenna modes visible along the propagation axis.

An important concern in alignment of THz time-domain spectrometers is a possibility to adjust it in a convenient way without employment of focusing or collimating optics. In what follows, it is illustrated that Ti-μbolometer can successfully be used to explore radiation patterns or quality of spatial modes without hyperhemispherical lens. The spatial beam characteristics (a scan in the x−y plane) recorded with 0.7 THz Ti-μbolometer without lens and System 1 are shown in Figure 6. As one can see, three lobes are still captured in a scanning area of 7 × 7 mm${}^{2}$ although signal-to-noise ratio was about 3.3. Careful inspection allows noting that these three constituents of the spatial mode do not diverge while going along *z*—direction indicating good alignment of the optical system. As is known, about 21 % of the incident power passes through silicon lens [25], on the other hand, without the lens radiation is dispersed over the large angles, therefore, only a small fraction of the total emitted power can be registered. Despite this fact, room temperature Ti-μbolometers sensitivity of 200 kV/W including amplification circuit and NEP of 20 pW/$\sqrt{\mathrm{Hz}}$ [23] is sufficient to register weak signal from photoconductive THz emitter even though the output beam is not effectively concentrated by the hyperhemispherical lens around the optical axis.

### 3.4. Time-Domain Versus Solid State-Based Solutions

Quality of the spatial beam profile and their control as well as propagation properties of the wideband THz beams was one of the prime interests since invention of THz time-domain spectrometers. Special attention was given to an angular distribution of the radiation emitted from a terahertz antenna system equipped with a truncated spherical silicon lens [25], where THz pulses were recorded by moving the photoconductive antenna in space and determining the intensity of the desired frequency at every point. Despite time consuming and complex experiments, authors successfully reconstructed THz beam profiles and found excellent agreement with theoretical estimates. To increase the THz emission power, antenna-biased system were investigated [27]; to optimise spectrometer operation, practical procedures for measuring the beam quality of the wideband THz radiation were introduced [28]. It is worth mentioning several other experimental techniques to evaluate THz-TDS beam profiles, for instance, electro-optical sampling using non-linear crystal [29], variable size aperture scanning [30] or fixed aperture raster scanning and Hartmann test [31]. These approaches allow deeper insight in both temporal and spatial THz electric field evolution, as well as emission pattern determination. However, they usually suffer of either diffraction limited resolution, either extensive time requirements or low detected signal levels. For example, variable aperture scanning usually experiences edge diffraction limited spatial resolution, at small aperture diameters in particular. Moreover, it measures only the integrated spectral power and is not suitable for a study of the wideband radiation since the low frequency portion can be filtered out at small apertures. Fixed aperture raster scanning features relatively low signal-to-noise ratio; Hartmann test technique is time consuming as it requires collecting at least twice as much data as fixed aperture raster scanning [31].

In real implementation schemes of THz-TDS spectrometers or THz imaging systems, beam quality control, pattern monitoring and optical alignment require much simpler and convenient-in-use solutions. Preference to use incoherent solid-state solutions in THz-TDS beam imaging exhibits lifted diffraction and signal level restrictions, as well as reduced recording time. Moreover, the devices are compact and reliable. As elegant examples can serve employment of CMOS-based field-effect transistors for THz autocorrelators development [32], coupled-charge device camera to measure spot size of the THz pulse generated by optical rectification in a mosaic organic crystal [33] or usage of high-speed sampling coupled to CMOS devices for hyperspectral imaging in THz time-domain system [34].

The presented study demonstrates that Ti-μbolometers can also be important part of the family bringing together solid-state-based sensors for precise and convenient incoherent validation of THz-TDS radiation patterns; it can serve for alignment aims, spatial modes quality control, for waist and divergence estimation even without additional focusing or collimating optics.

## 4. Conclusions

Lensless titanium-based antenna coupled microbolometers operating at room temperature are demonstrated for the precise monitoring of radiation pattern and spatial mode profiles control in terahertz time-domain spectroscopy systems. To provide comprehensive picture, two different THz-TDS systems and three Ti-μbolometers with individual antenna designs—narrowband dipole antenna for 0.3 THz, 0.7 THz and a log-periodic antenna for THz wideband detection—are used in the investigation.The radiation patterns, spatial mode control, and explicit beam evolution along the propagation axis were examined, polarization-sensitive properties were revealed under different THz emitter power range. To illustrate the beam quality control, the beam waist of 1.52 mm and divergence of 0.045 rad were estimated from the far-field measurements at 0.7 THz. It was shown that the Ti-μbolometers exhibiting room temperature sensitivity values of 200 kV/W, noise-equivalent power of 20 pW/$\sqrt{\mathrm{Hz}}$, and the response time in the 5 μs range are sensitive and fast enough to monitor precisely coherent THz emitters in optoelectronic time-domain systems even without any focusing and collimating optics elements.

## Figures and Tables

**Table 1:** The main performance and antenna geometrical parameters of the resonant (0.3 THz and 0.7 THz) and the wideband detectors. Note the same NEP for both resonant antenna-coupled devices. Presented sensitivity values are of Ti-μbolometer itself, without electronics.

Detector Type	Sensitivity [V/W]	NEP [pW/$\sqrt{\mathrm{Hz}}$]	*L* [μm]	*W*[μm]	ϕ [deg]
0.3 THz	1000	20	674	16	–
0.7 THz	1000	20	247	39	–
Wideband	50	60	1042	–	45

## Data Availability

Not applicable.

## Abbreviations

The following abbreviations are used in this manuscript:

THz	Terahertz
THz-TDS	Terahertz time-domain spectroscopy
μbolometers	microbolometers
LT-GaAs	Low temperture gallium arsenide
Ti	Titanium
NEP	Noise Equivalent Power

## References

[1] Cheung K., Auston D. (1986). A novel technique for measuring far-infrared absorption and dispersion. Infrared Phys..

[2] Neu J., Schmuttenmaer C.A. (2018). Tutorial: An introduction to terahertz time domain spectroscopy (THz-TDS). J. Appl. Phys..

[3] Abina A., Puc U., Jeglič A., Zidanšek A. (2016). Structural characterization of thermal building insulation materials using terahertz spectroscopy and terahertz pulsed imaging. NDT E Int..

[4] Xie L., Yao Y., Ying Y. (2014). The application of terahertz spectroscopy to protein detection: A review. Appl. Spectrosc. Rev..

[5] Son J.H., Oh S.J., Cheon H. (2019). Potential clinical applications of terahertz radiation. J. Appl. Phys..

[6] Sun Q., He Y., Liu K., Fan S., Parrott E.P., Pickwell-MacPherson E. (2017). Recent advances in terahertz technology for biomedical applications. Quant. Imaging Med. Surg..

[7] Trofimov V.A., Varentsova S.A. (2019). A possible way for the detection and identification of dangerous substances in ternary mixtures using thz pulsed spectroscopy. Sensors.

[8] Puc U., Abina A., Rutar M., Zidanšek A., Jeglič A., Valušis G. (2015). Terahertz spectroscopic identification of explosive and drug simulants concealed by various hiding techniques. Appl. Opt..

[9] Miyagawa K., Nagai M., Yamashita G., Ashida M., Kim C., Akiyama H., Kanemitsu Y. (2018). Quantitative monitoring of the internal field in the depletion layer of a GaAs-based solar cell with terahertz radiation. Appl. Phys. Lett..

[10] Minkevičius L., Suzanovičiene R., Balakauskas S., Molis G., Krotkus A., Valušis G., Tamošiunas V. (2012). Detection of tab wire soldering defects on silicon solar cells using terahertz time-domain spectroscopy. Electron. Lett..

[11] Afsah-Hejri L., Hajeb P., Ara P., Ehsani R.J. (2019). A Comprehensive Review on Food Applications of Terahertz Spectroscopy and Imaging. Compr. Rev. Food Sci. Food Saf..

[12] Karaliūnas M., Nasser K.E., Urbanowicz A., Kašalynas I., Bražinskienė D., Asadauskas S., Valušis G. (2018). Non-destructive inspection of food and technical oils by terahertz spectroscopy. Sci. Rep..

[13] Wan M., Healy J.J., Sheridan J.T. (2020). Terahertz phase imaging and biomedical applications. Opt. Laser Technol..

[14] Aghamiri N.A., Huth F., Huber A.J., Fali A., Hillenbrand R., Abate Y. (2019). Hyperspectral time-domain terahertz nano-imaging. Opt. Express.

[15] Puc U., Abina A., Jeglič A., Zidanšek A., Kašalynas I., Venckevičius R., Valušis G. (2018). Spectroscopic Analysis of Melatonin in the Terahertz Frequency Range. Sensors.

[16] Kašalynas I., Venckevičius R., Tumonis L., Voisiat B., Seliuta D., Valušis G., Račiukaitis G. (2013). Reflective terahertz imaging with the TEM01 mode laser beam. Appl. Opt..

[17] Minkevičius L., Jokubauskis D., Kašalynas I., Orlov S., Urbas A., Valušis G. (2019). Bessel terahertz imaging with enhanced contrast realized by silicon multi-phase diffractive optics. Opt. Express.

[18] Jokubauskis D., Minkevičius L., Karaliūnas M., Indrišiūnas S., Kašalynas I., Račiukaitis G., Valušis G. (2018). Fibonacci terahertz imaging by silicon diffractive optics. Opt. Lett..

[19] Oden J., Meilhan J., Lalanne-Dera J., Roux J.F., Garet F., Coutaz J.L., Simoens F. (2013). Imaging of broadband terahertz beams using an array of antenna-coupled microbolometers operating at room temperature. Opt. Express.

[20] Oda N. (2010). Détecteur matriciel de type bolométrique à température ambiante et camera vidéo pour l’imagerie térahertz. Comptes Rendus Phys..

[21] Trontelj J., Sešek A. (2016). Electronic terahertz imaging for security applications. Terahertz Rf Millimeter-Submillimeter-Wave Technol. Appl. IX.

[22] Kašalynas I., Venckevičius R., Minkevičius L., Sešek A., Wahaia F., Tamošiūnas V., Voisiat B., Seliuta D., Valušis G., Švigelj A. (2016). Spectroscopic terahertz imaging at room temperature employing microbolometer terahertz sensors and its application to the study of carcinoma tissues. Sensors.

[23] Minkevičius L., Qi L., Siemion A., Jokubauskis D., Sešek A., Švigelj A., Trontelj J., Seliuta D., Kašalynas I., Valušis G. (2020). Titanium-based microbolometers: Control of spatial profile of terahertz emission in weak power sources. Appl. Sci..

[24] Sešek A., Kašalynas I., Žemva A., Trontelj J. (2017). Antenna-coupled Ti-microbolometers for High-sensitivity Terahertz Imaging. Sens. Actuators A Phys..

[25] Jepsen P.U., Keiding S.R. (1995). Radiation patterns from lens-coupled terahertz antennas. Opt. Lett..

[26] Van Rudd J., Johnson J.L., Mittleman D.M. (2001). Cross-polarized angular emission patterns from lens-coupled terahertz antennas. J. Opt. Soc. Am. B.

[27] Jepsen P.U., Jacobsen R.H., Keiding S.R. (1996). Generation and detection of terahertz pulses from biased semiconductor antennas. J. Opt. Soc. Am. B.

[28] Bitman A., Lumer Y., Moshe I., Zalevsky Z. (2012). Characterization of spectrally broadband terahertz beam propagation. J. Opt. Soc. Am. B.

[29] Gürtler A., Winnewisser C., Helm H., Uhd Jepsen P. (2000). Terahertz pulse propagation in the near field and the far field. J. Opt. Soc. Am. A.

[30] Molloy J.F., Naftaly M., Dudley R.A. (2011). Characterisation of Terahertz Beam Profile and Propagation through Complex Quasi-Optic Systems. Int. J. Terahertz Sci. Technol..

[31] Molloy J.F., Naftaly M., Dudley R.A. (2013). Characterization of Terahertz Beam Profile and Propagation. IEEE J. Sel. Top. Quantum Electron..

[32] Ikamas K., Nevinskas I., Krotkus A., Lisauskas A. (2018). Silicon field effect transistor as the nonlinear detector for terahertz autocorellators. Sensors.

[33] Chefonov O.V., Ovchinnikov A.V., Agranat M.B., Stepanov A.N. (2019). Terahertz beam spot size measurements by a CCD camera. Opt. Lett..

[34] Tsubouchi M., Nagashima K. (2020). High-speed terahertz color imaging using a 100 kHz line scan camera. Opt. Express.

